# Poor adherence is a major barrier to the proper treatment of cutaneous leishmaniasis: A case-control field assessment in Iran

**DOI:** 10.1016/j.ijpddr.2022.11.006

**Published:** 2022-12-05

**Authors:** Mehdi Bamorovat, Iraj Sharifi, Setareh Agha Kuchak Afshari, Ali Karamoozian, Amirhossein Tahmouresi, Amireh Heshmatkhah, Ehsan Salarkia, Ahmad Khosravi, Maryam Hakimi Parizi, Maryam Barghi

**Affiliations:** a Leishmaniasis Research Center, Kerman University of Medical Sciences, Kerman, Iran; b Medical Mycology and Bacteriology Research Center, Kerman University of Medical Sciences, Kerman, Iran; c Research Center for Modeling in Health, Institute for Futures Studies in Health, Kerman University of Medical Sciences, Kerman, Iran; d Dadbin Health Clinic, Kerman University of Medical Sciences, Kerman, Iran; e Department of Biochemistry, Faculty of Veterinary Medicine, Shiraz University, Shiraz, Iran

**Keywords:** Poor adherence, Cutaneous leishmaniasis, Major barrier, Treatment, Iran

## Abstract

Leishmaniasis is an overlooked, poverty-stricken, and complex disease with growing social and public health problems. In general, leishmaniasis is a curable disease; however, there is an expansion of unresponsive cases to treatment in cutaneous leishmaniasis (CL). One of the effective and ignored determinants in the treatment outcome of CL is poor treatment adherence (PTA). PTA is an overlooked and widespread phenomenon to proper *Leishmania* treatment. This study aimed to explore the effect of poor adherence in unresponsiveness to treatment in patients with anthroponotic CL (ACL) by comparing conventional statistical modalities and machine learning analyses in Iran. Overall, 190 cases consisting of 50 unresponsive patients (case group), and 140 responsive patients (control group) with ACL were randomly selected. The data collecting form that included 25 queries (Q) was recorded for each case and analyzed by R software and genetic algorithm (GA) approaches. Complex treatment regimens (Q11), cultural and lay views about the disease and therapy (Q8), life stress, hopelessness and negative feelings (Q22), adverse effects of treatment (Q13), and long duration of the lesion (Q12) were the most prevalent significant variables that inhibited effective treatment adherence by the two methods, in decreasing order of significance. In the inherent algorithm approach, similar to the statistical approach, the most significant feature was complex treatment regimens (Q11). Providing essential knowledge about ACL and treatment of patients with chronic diseases and patients with misconceptions about chemical drugs are important issues directly related to the disease's unresponsiveness. Furthermore, early detection of patients to prevent the long duration of the disease and the process of treatment, efforts to minimize side effects of treatment, induction of positive thinking, and giving hope to patients with stress and anxiety by medical staff, and family can help patients adhere to the treatment.

## Highlights

• The study aimed to explore the effect of poor adherence in unresponsive CL cases.
• The data collecting form that included 25 queries (Q) was recorded for each case.
• Conventional statistical modalities and machine learning analyses were used.
• The most significant feature was complex treatment regimens (Q11).
• Providing essential knowledge about the treatment of CL cases is an important issue.

## Graphical abstract

**Figure:**
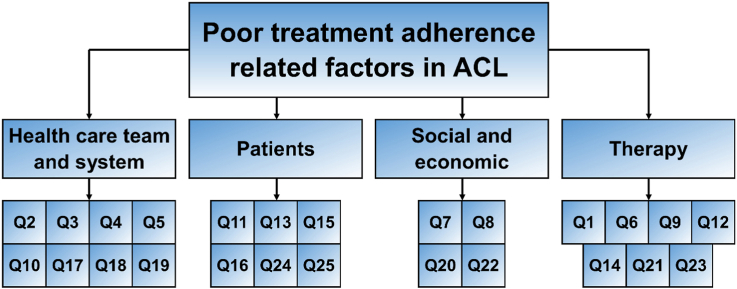

## 1 Introduction

Leishmaniasis is an overlooked, poverty-stricken, and complex tropical disease with increasing social and community health problems (Bailey et al., 2017; WHO, 2016). It is endemic in over 100 countries and territories and is focused primarily on low-income people in the affected tropical areas (WHO, 2019; WHO, 2017). Leishmaniasis presents diverse clinical and epidemiological forms depending on the parasite species and spatial areas (Bamorovat et al., 2021a). The most prevalent form of leishmaniasis is cutaneous leishmaniasis (CL) (Alvar et al., 2012; Bailey et al., 2017). Generally, the annual global incidence of CL is estimated to be between 0.7 and 1 million new cases and this rate in Iran is 15,000 to 20,000 (WHO, 2019; Shirzadi et al., 2015).

In Iran, CL presents itself in two scientific forms: anthroponotic CL (ACL), familiar as urban-type (dry-form) produced by *Leishmania tropica*, and zoonotic CL (ZCL) as rural-type (wet-form) induced by *L. major*. In a usual ZCL form the primary lesion usually progresses into an ulcer which heals naturally after several weeks or months, resulting in a permanent scar. In contrast, ulceration is not a significant characteristic of the ACL type due to hyperkeratosis in the domain of lesions and low antigenicity of the causative agent (Bamorovat et al., 2018, 2019, 2021a; Rostami et al., 2013; Saghafipour et al., 2013).

Meglumine antimoniate (MA, Glucantime®) is the choice drug of CL injected as intramuscular (for 3–4 weeks) and intralesionally (once a week for 8–12 weeks along with biweekly cryotherapy) (Shirzadi and Gouya, 2012; WHO, 2014). In contraindication of the standard drug (MA) of CL, other medicines such as amphotericin B deoxycholate, lipid formulations of amphotericin B, miltefosine, paromomycin, fluconazole, ketoconazole, and itraconazole and also combination therapy, can be applied (Firooz et al., 2020). Table 1 shows to summarize the usual drugs used to treat CL cases in Iran with their dosage and routes.

**Table 1:** Conventional drugs to treat cutaneous leishmaniasis cases in Iran.

Type of treatment	Route of treatment	Dosage, timing, and duration
Meglumine antimoniate (MA)	Intramuscular	20 mg/kg every day for 21 days
MA + Cryotherapy	Intralesional	20 mg/kg every week for 12 weeks along with biweekly cryotherapy
Paromomycin	Topical ointment	Paromomycin 15% ointment with methyl benzethonium chloride 12% twice a day for 20 days

Efforts to produce efficacious vaccines against leishmaniasis have so far been ineffective, however, many clinical and field trials of inactivated, killed and live vaccines have been carried out worldwide (Khamesipour et al., 2005). In Uzbekistan, one live vaccine including a live virulent *L. major* combined with an inactive *Leishmania* parasite was used (Khamesipour et al., 2005). Furthermore, leishmanization is the inoculation of live virulent *Leishmania*, and it has been used in various countries for about 60 years (Khamesipour et al., 2005). Generally, leishmaniasis is a curable disease (WHO, 2016); however, the expansion of insensitive CL patients is also related to manifold determinants such as clinical, demographical, and environmental factors, PTA, host's immune response, and the intrinsic makeup of the parasite (Bamorovat et al., 2021b). In Iran, some counties such as Bam, Kerman, and Mashhad, the rate of unresponsive ACL cases was reported to be 11–12% (Aflatoonian et al., 2019; Hadighi et al., 2006; Karvar et al., 2016; Laffitte et al., 2005). The World Health Organization (WHO) defines medicine adherence as " the degree to which a patient's drug use matches the recommended regimen” (behavior that follows a healthcare provider's agreed-upon guidelines) (WHO, 2003). Poor adherence to planned schedules can result in serious health outcomes. Some studies have stated that poor therapy adherence among cases with various diseases averages as much as 50% in developed nations, while this is significantly higher in underdeveloped countries (Haynes et al., 2002).

The rate of PTA raises as the burden of long-lasting chronic diseases progresses globally. Some associated factors concomitantly influence adherence. The competence of patients to follow treatment regimens is frequently associated with multiple barriers, usually linked to various characteristics of the complication. These additional factors comprise the socioeconomic, the health personnel, the features of the disease, therapies, and patient-connected aspects. Resolving such comorbidities is essential if patients’ adherence to treatment can be enhanced (WHO, 2003). Economic poverty is one of the main factors influential in neglected treatment adherence, especially in leishmaniasis (Farmer, 1999; Robinson, 1997; Timmreck et al., 1993).

Reports indicate that the CL cases treated with fewer doses of medicine during the treatment (fewer quantities of drug) or who had commenced therapy late had a greater risk of creating an unresponsive form than those who started treatment shortly following the onset of the disease (Bamorovat et al., 2018). The lack of active-case finding approaches and consciousness among the populations, random treatments, and lack of health facilities were all linked with late CL detection (Aflatoonian et al., 2019). Therefore, the therapy started at a maximum of 4 months after that, and, in this condition, the treatment process would be more complex and challenging. Inadequate or uneven CL treatment can occasionally cause amplified drug resistance to *Leishmania* (Natera et al., 2007; Ouellette et al., 2004)*.*

As humans are the sole reservoir host, the main problem in ACL is the development of unresponsiveness to treatment; therefore, it is necessary to know the influential factors such as PTA, which possibly play a pivotal role in the cure rate (Bamorovat et al., 2021b). PTA has a deeply undesirable influence on the consequence of chemotherapy (Aflatoonian et al., 2019; Cohen and Saran, 2018; WHO, 2003; Umeokonkwo et al., 2019; Uranw et al., 2013). The study results exposed that a considerable number of patients did not obey their doctor's instructions, so treatment observance was a significant challenge for patients and healthcare workers (Aflatoonian et al., 2019).

To our knowledge, so far, no study has been carried out to evaluate the obedience of patients to treatment against CL. Nobody knows the dimension of treatment adherence to this perplexing disease. Therefore, this study aimed to explore the effect of poor adherence factors in unresponsiveness to treatment relative to responsive ones in patients with ACL by comparing conventional statistical modality and machine learning analyses in a high-burden focus in Iran.

## 2 Methods

### 2.1 Design and study site

This investigation was carried out as case-control research in Kerman district, Kerman province, between February 2020 and October 2021, Iran's largest province and 1000 km southeast of Tehran. Kerman district is a well-recognized focus of ACL in the country (Sharifi et al., 2015). Some grounds such as geographical and environmental conditions and also urbanization especially in suburbs of the city provide numerous risk factors and appropriate breeding surroundings for proliferating phlebotomine sandflies and reservoir hosts, leading to an excessive number of CL patients in a remote part of Iran.

### 2.2 Sample collection

This study was conducted on ACL patients treated with MA as intralesional along with cryotherapy and systemically. Patients with refractory and responsiveness to treatment were randomly selected as case and control groups in a high-burden focus with ACL in Kerman. A total of 190 patients were studied, of which 50 patients did not respond to treatment (unresponsive patients, case group), and 140 patients responded to treatment (responsive patients, control set). Of these patients, n = 105 (55.3%) were females (n = 31 unresponsive and n = 74 responsive patients), and n = 85 (44.7%) were males (n = 19 unresponsive and n = 66 responsive patients). Dadbin Health Clinic, the principal referral center for CL management, accommodated the current study. School of Medicine and Leishmaniasis Research are closely linked to where the study was conducted. Also, researchers from different fields assessed the content in terms of its accuracy. The studied questionnaire queries are shown in Table 2.

**Table 2:** The main variables/questions are assessed in the data-collecting form.

No	Question	Answer	
Q1	Long distance from treatment setting	>40 km □	<40 km □
Q2	Lack of knowledge of health professionals about pain management	Yes□	No□
Q3	Poor delivery of care-education to the patient	Yes□	No□
Q4	Poor delivery of care education to family and caregivers	Yes□	No□
Q5	Good relationship between patient and health personnel	Yes□	No□
Q6	Family support	Yes□	No□
Q7	Poverty and low socioeconomic status	Good□	Poor□
		Moderate□	
Q8	Cultural and lay beliefs about illness and treatment	Yes□	No□
Q9	Recent immigrants	Yes□	No□
Q10	Poorly developed health services	Yes□	No□
Q11	Complex treatment regimens	Yes□	No□
Q12	Long duration of lesion	>4 month □	<4 month □
Q13	Adverse effects of treatment	Yes□	No□
Q14	Absence of treatment for any reason	Yes□	No□
Q15	Inadequate treatment doses	Yes□	No□
Q16	Self-management of disease and treatment	Yes□	No□
Q17	Mistake in initial diagnosis	Yes□	No□
Q18	Lack of clear instructions from health professionals	Yes□	No□
Q19	Lack of knowledge and training for health care providers on managing chronic diseases	Yes□	No□
Q20	Unstable living conditions	Yes□	No□
Q21	Forgetfulness	Yes□	No□
Q22	Life stress, hopelessness and negative feelings	Yes□	No□
Q23	Active participation in monitoring	Yes□	No□
Q24	Believe of lack and poor effective drug	Yes□	No□
Q25	Irregular treatment	Yes□	No□

Critical facts about the study were stated to the volunteer patients or their parents. Through interviews, the assessors made sure that the members or their protectors are well-aware of the queries. Participants were excluded from the research if they refused to respond to some questions or answered them with vagueness and inattention during the meetings.

### 2.3 Ethical considerations

The Ethics Committee of the Kerman University of Medical Sciences approved the project (ethics no. IR.KMU.REC.1398.215, and contract no. 98000240). At first, many eye-to-eye assemblies and meetings with the patients, parents, and public health authorities were arranged. The primary aim and the possible benefits were clarified. For each patient, a standard informed consent form was completed and obtained. Patients with CL volunteered to participate in the trial. All information was kept confidential.

### 2.4 Statistical approach

For data analysis, we used R software (version 4.1.2). In the present study, dominance analysis was used to determine the most influential variables in the development of unresponsive forms. Four models were used, including McFadden, Cox & Snell, Negelkerke, and Estrella coefficients of determination (R2). Furthermore, Bootstrapping analysis was used to evaluate the robustness of the results.

### 2.5 Metaheuristic approach

A metaheuristic-based approach applied a genetic algorithm (GA) to find the best-established features in our dataset. The classification accuracy is the fitness function employed in GA, and the subset of features that produce the best accuracy gets higher ranks. After searching for the best set of attributes, they could be identified as the most significant features. The confusion matrix and receiver operating characteristic (ROC) plot with the area under this curve (AUC) were presented. In the GA algorithm, the average of 30 separate runs was considered. The dataset was separated into two subsets: training (80%) and test (20%). Moreover, GA's number of populations, generation, crossover rate, and mutation rate were set to 120, 10, 30, and 30, respectively. All the computational processes were run on MATLAB 2021 software.

## 3 Results

### 3.1 Statistical results

The highest value based on the four indicators is the variable Q11 (complex treatment regimens) indicating that the variable Q11, more than other variables, justified the changes in the response variable in the model. In other words, this variable has more effect on the response mutable than other ones. There are “complex treatment regimens” in CL patients along with chronic diseases such as diabetes, hypertension, tuberculosis, HIV/AIDS, allergies, and addiction that simultaneously get infected with CL. Hence, they take several treatment regimens due to multiple diseases at the same time. These patients usually do not receive the CL treatment completely and regularly because they take many drugs and put the treatment of CL as the next priority.

**Fig. 1:**
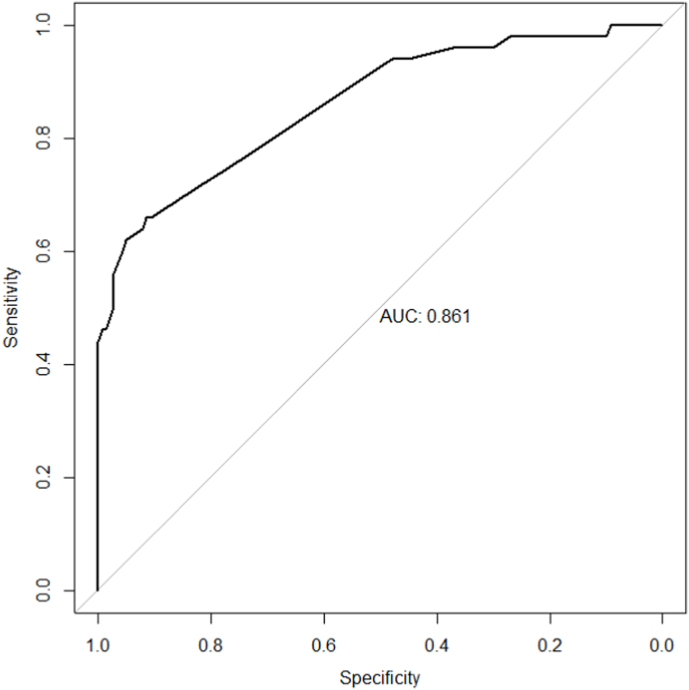
Confusion matrix and receiver operating characteristic (ROC) curve in statistical approach.

In successive positions, the variables adverse effects of treatment (Q13), absence of information from health experts about pain management (Q2), life stress, hopelessness and negative feelings (Q22), long duration of the lesion (Q12), cultural and lay beliefs about illness and treatment (Q8), a mistake in initial diagnosis (Q17) and forgetfulness (Q21) were the other most influential components, respectively. Table 3 shows the highest value based on the four indicators. Also, Fig. 1 specifies the confusion matrix and receiver operating characteristic (ROC) curve. In this statistical approach, other analyzed variables/questions were not significant.

**Table 3:** The significant values are based on the McFadden, Cox & Snell, Negelkerke, and Estrella coefficients of determination (R2) among all variables.

Variable	McFadden			Cox & Snell			Negelkerke			Estrella		
	R2	SE	bias	R2	SE	bias	R2	SE	bias	R2	SE	bias
Q2	0.026	0.017	0.0039	0.023	0.012	0.0022	0.033	0.022	0.0073	0.029	0.022	0.0083
Q8	0.012	0.004	0.0001	0.012	0.004	0.0003	0.017	0.006	0.0001	0.014	0.005	0.0003
Q11	0.198	0.047	0.0021	0.181	0.038	0.0041	0.264	0.061	0.0130	0.220	0.050	0.0002
Q12	0.013	0.013	0.0046	0.013	0.013	0.0065	0.02	0.018	0.0061	0.014	0.015	0.0057
Q13	0.119	0.048	0.0043	0.112	0.039	0.0019	0.163	0.052	0.0072	0.133	0.046	0.0046
Q17	0.005	0.009	0.0041	0.005	0.006	0.0021	0.008	0.014	0.0065	0.006	0.010	0.0052
Q21	0.005	0.008	0.0037	0.004	0.007	0.0045	0.006	0.013	0.0060	0.005	0.010	0.0058
Q22	0.017	0.015	0.0045	0.015	0.015	0.0064	0.023	0.020	0.0027	0.019	0.015	0.0020

R2: coefficients of determination.

SE: standard error.

### 3.2 Genetic algorithm results

**Fig. 2:**
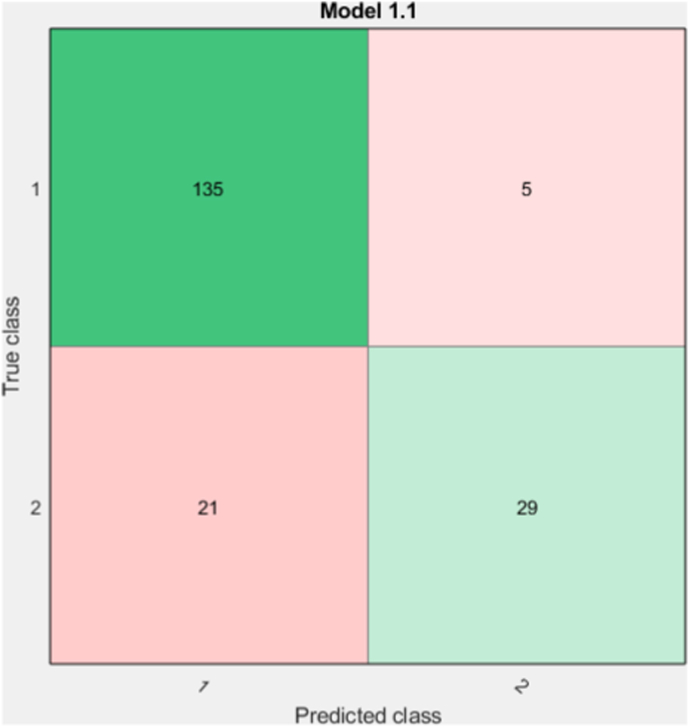
The confusion matrix of the selected features with support vector machine (SVM) classifier, class 1: Responsive, and class 2: Unresponsive.

**Fig. 3:**
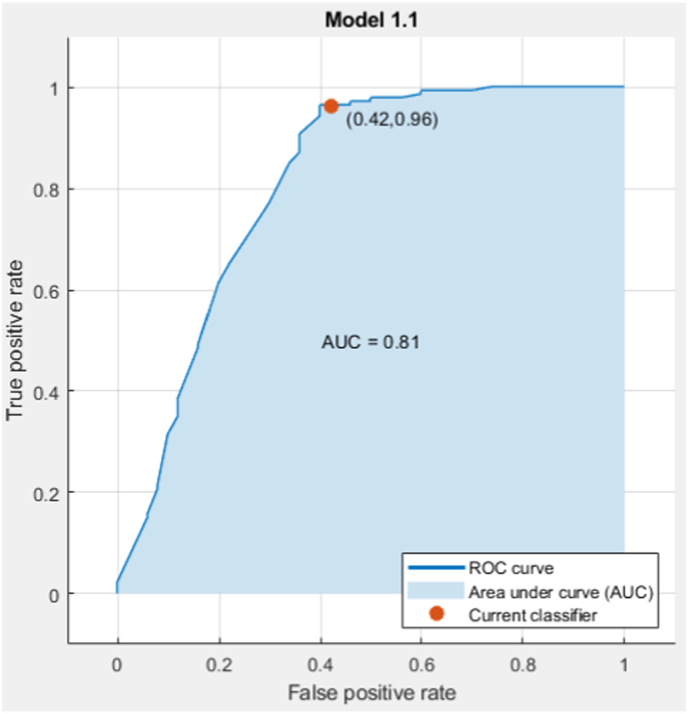
The receiver operating characteristic (ROC) plot curve of selected features via support vector machine (SVM) classifier and indicated area under the curve (AUC).

Table 4 lists the selected attributes, the algorithm's calculation time, the optimal support-vector machine (SVM) kernel for the model, and the algorithm's accuracy. Fig. 2, Fig. 3 specify the confusion matrix and receiver operating characteristic (ROC) curve. Moreover, in Table 5 the rate of positive predictive value (PPV), false discovery rate (FDR), true-positive rate (TPR), and false-negative rate (FNR) indicate the classifier status, which are the leading performance parameters of the classification problem.

**Table 4:** Overall accuracy and time-efficacy of feature selection in a genetic algorithm (GA).

Algorithm	Accuracy	Elapsed time per one time run based on seconds	Best SVM kernel	Number of selected attributes
GA	86.3%	6.7	Linear SVM	8 (19,22,24,11,13,8,14,12)

**Table 5:** The classification problem parameters for 2 classes (class 1: Responsive and class 2: Unresponsive).

Classifier measurements	Class 1: Responsive	Class 2: Unresponsive
PPV	87%	85%
FDR	13%	15%
TPR	96%	58%
FNR	4%	42%

PPV: positive predictive value.

(FDR): false discovery rate.

TPR: true-positive rate.

FNR: false-negative rate.

Removing the selected features can be applied to indicate the essential feature in this classification problem. In this regard, the classification problem will be solved with the SVM model by removing one of the attributes in Table 6.

**Table 6:** Removing the features one by one to indicate the most significant attribute.

Features	Q19	Q22	Q24	Q11	Q13	Q8	Q14	Q12
Accuracy	86.3%	86.3%	85%	81%	83.7%	86%	86.4%	86%

In Table 6, the most significant feature was Q11 (complex treatment regimens) due to the accuracy of the classifier has reduced most, and it had the most impact on the classification problem. In the following positions, the variables adverse effects of treatment (Q13), belief of lack and poor effective drug (Q24), cultural and lay beliefs about disease and treatment (Q8), long duration of the lesion (Q12), lack of knowledge and training for healthcare providers on managing chronic diseases (Q19), life stress, hopelessness and negative feelings (Q22), nonattendance of treatment for any reason (Q14) were the most influential variables, respectively. Also, in the genetic algorithm model, other variables/questions were not significant.

### 3.3 The common significant variables in both analyses

It is worth noting that in genetic algorithm analysis, similar to statistical analysis, the most significant feature was Q11. Table 7 displays the significant variables in the two analysis methods and the common significant variables.

**Table 7:** Displays the significant variables in the two analysis methods and the common significant variables.

Common significant variables by two methods	Significant variables by statistical approach	Significant variables by genetic algorithm
**Q8**	Q8	Q8
**Q11**	Q11	Q11
**Q12**	Q12	Q12
**Q13**	Q13	Q13
**Q22**	Q22	Q22
	Q2	Q14
	Q17	Q19
	Q21	Q24

## 4 Discussion

**Fig. 4:**
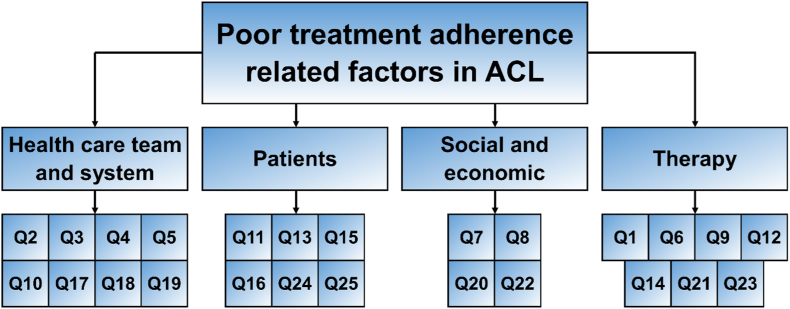
Factors affecting adherence to treatment for CL.

Factors disturbing faithfulness to treatment for diseases such as CL and interventions for refining them included socioeconomic, healthcare/health system, therapy, and patient-related factors (WHO, 2003). Fig. 4 shows the categories of related factors for adherence to treatment in CL in this study.

In this study, the complex treatment regimen (Q11) had the most impact and significance on both methods' response variables (PTA). The expected significant variables in the two approaches were complex treatment regimens, cultural and lay beliefs about the disease and treatment, life stress, hopelessness and negative feelings, adverse effects of therapy, and the long duration of the lesion.

The problem of poor adherence is of great concern to all stakeholders in the healthcare system since the burden of sickness in the population has moved to chronic diseases. This fact increases the risk of poor adherence to treatment with the complex regimen (Q11) and long duration of treatment (Q12), and both complex and lengthy behavior is characteristic of chronic illnesses. Patients accompanying chronic diseases infected with leishmaniasis at the same time should include a new regimen of therapy on their schedule and their previous regime of therapy. There is a solid indication that numerous patients having chronic diseases, including hypertension, diabetes, asthma, and immunodeficient individuals, have trouble following their proposed schedules. The primary cause of unsatisfactory clinical benefit is low adherence (Dunbar-Jacob et al., 2000; Rybacki, 2002). This fact results in medical and psychological difficulties, reduced patient quality of life, and a waste of healthcare resources. Altogether, these direct sequels weaken the capability of healthcare systems worldwide to accomplish population health goals (Dunbar-Jacob et al., 2000; WHO, 2003).

Also, in statistical analysis, Aflatoonian et al. (2019) demonstrated that ACL cases with lesions lasting more than 4 months before starting the first treatment (long duration of treatment, Q12) who had partial treatment adherence were meaningfully linked with unresponsiveness to the therapy (Aflatoonian et al., 2019). Some other patients fail treatment due to traditional drugs and other therapies, and the primary treatment is due to cultural and lay opinions about disease and treatment (Q8). These patients often cause them to abandon the conventional treatment (standard gold treatment) due to the pain caused by the injection of MA and continue the use of traditional drugs (WHO, 2003). Opinions about drugs and awareness of chemical therapy and infections vary among different societies (Al Saeedi et al., 2003). Findings of a study in Suriname indicated that CL patients apply potentially damaging non-biomedical substances such as battery acid, lead, gasoline, chlorine, insecticides, and herbicides to heal their lesions (Ramdas, 2012). Psychological suffering has also been revealed to disturb adherence. Depression, anxiety, and how individuals succeed in stress are among the most significant forecasters of adherence (Chesney et al., 2000; Murphy et al., 2001; Paterson and Singh, 2001). ‘Hopelessness and negative feelings’ (Q22) can decrease motivation to meditate for self and influence a patient's ability to follow complex orders (Murphy et al., 2001). These outcomes are comparable to investigations on other chronic circumstances that have confirmed an association between adherence and depression (Dunbar-Jacob et al., 1995).

Adherence issues are reported in all cases when self-administration of therapy is necessary, independent of the kind of disease, disease severity, or accessibility to health providers, according to research findings (WHO, 2003). While it may appear to be a simple problem, adherence issues are caused by a variety of causes. Although some of these elements are connected to the patient, the features of the disease and its treatment, as well as the characteristics of the healthcare system and service delivery, all have a role. As a result, adherence issues have largely been disregarded by health stakeholders, who have received little direct and systematic intervention as a result (WHO, 2003). The issue of noncompliance has received a lot of attention, but it has been largely ignored in the traditional delivery of primary care health services. Despite a large body of information, efforts to address the problem have been disjointed, with few exceptions, failing to integrate the potential contributions of other health fields. A more extensive pledge to a multidisciplinary method is crucial to make improvements in this part. This will want joint functions from health authorities, investigators, health designers, and decision-makers.

Despite evidence of the conflict, there remains a predisposition to emphasize patient-linked factors as the reasons for difficulties with adherence, the relative abandonment of providers, and health system-related causes. These latter factors devise the healthcare setting in which patients obtain care and noticeably affect adherence. Adherence is connected to how people judge individual needs compared to their fears about possible harmful effects (Horne and Weinman, 1999). Horne et al. projected a simple necessity-concerns basis to assist physicians in provoking and addressing essential opinions that impact patients' cohesion to drugs. Requirement beliefs and worries assess summaries of the therapy's salience, probably expenses, and benefits or advantages and disadvantages (Horne, 1999). The significances of fractional adherence to prolonged treatments are unfortunate health results and augmented healthcare expenses. Adherence is a principal element of the efficacy of therapy (Cohen and Saran, 2018; Uranw et al., 2013) because inconsistency reduces optimal medical advantage (Dunbar-Jacob et al., 2000; Sarquis et al., 1998).

Improving physicians' obedience also promotes patients' security. Patients envisage numerous potentially serious hazards if health references are not trailed as arranged, including more durable relapses, enhanced risk of dependence, amplified risk of self-restraint and rebound effect, greater risk of emerging resistance to treatments, and enhanced risk of harmfulness. Improving faithfulness could be the best asset for combating chronic diseases successfully. For better-quality consequences, essential health services and health policy changes are vigorous. Deprived of a system that addresses the elements of adherence, progress in health skills will be unsuccessful in realizing the possibility of decreasing the effect of chronic disease. Admission to drugs is vigorous but inadequate to resolve the problem (WHO, 2003). Promoting the efficiency of adherence management could significantly impact the population's health relative to any development in precise medical treatments (Haynes et al., 2002).

The public belief that patients are exclusively in charge of their therapy is deceptive and frequently reproduces confusion about how other aspects disturb working-class performance and dimensions to follow their treatment. Adherence is a multidimensional spectacle strongminded by the interaction of several groups of issues. These contain the interplay of healthcare personnel and factors related to the system, patient, therapy, and socioeconomic.

Although the social and economic condition has not reliably been initiated to be a self-governing interpreter of adherence, in underdeveloped countries, poor rank may place patients in the situation to select between contending urgencies. Such urgencies often comprise demands to manage the inadequate resources accessible to meet the requirements of other household associates, such as parents or children for whom they care.

Comparatively, a negligible investigation has been focused on the influence of healthcare personnel and system-correlated factors on adherence. While an optimal patient healthcare provider association may advance adherence (Richardson et al., 2000), several factors negatively affect it. These comprise poorly developed health facilities with insufficient or non-existent repayment by health insurance programs, deprived drug delivery systems, and the absence of training and knowledge for healthcare workers on managing chronic diseases.

Patient-associated issues include resources, knowledge, tendency, understanding, behaviors, and patient prospects. Patients’ knowledge and beliefs regarding their disease, inspiration to accomplish it, self-assurance (self-efficacy) in their aptitude to engage in disease-management performances, prospects about the consequence of treatment, and the outcomes of poor adherence interrelate in ways not yet entirely recognized to impact adherence behavior.

In 2006, Rodríguez et al. demonstrated that PTA is the most important component of treatment failure. They showed that irregular treatment was a determinant in the treatment outcome of CL (RR: 1.85) (Rodrigues et al., 2006). A study performed on adult and child cases with CL in Colombia revealed that treatment adherence was weak (<90%) related to increased odds of unresponsive cases of CL (Castro et al., 2017). Good adherence to miltefosine treatment for VL in Nepal has been reported in 57.9% of patients. Treatment adherence was higher in cases who were well-informed about side effects rather than those that did not know (Uranw et al., 2013).

Results of other infectious diseases showed that good adherence increases the efficacy of interventions. Evidence from a randomized controlled trial in Uganda showed the impact of packaging and messaging on good adherence to malaria treatment (Cohen and Saran, 2018). A study in Ghana demonstrated improving adherence to malaria treatment for children using reform in treatment methods (Ansah et al., 2001). In infectious chronic diseases such as HIV, good adherence to therapies has been associated with the slower clinical development of the infection as well as fewer virological markers (Bangsberg et al., 2000; Umeokonkwo et al., 2019).

The limitation of the study was that Kerman, as the largest province, is a vast and diverse area with many rural communities located in remote areas. We found the assigned cases and selected them randomly to meet the pre-determined sample size. The main strength of the current study is the well-equipped Health Clinics associated with the Leishmaniasis Research Center, strong registry systems, robust infrastructures that manage the patients by experienced staff and physicians, and a well-organized team that works with suitable facilities for diagnosis and treatment. This study is unique since no similar study has already been carried out to explore the variable dimensions of such common prevailing barriers in proper treatment of CL with meglumine antimoniate, the first-line drug frequently used nationally and globally as well.

The findings represented that in the inherent algorithm approach, similar to the statistical approach, the most significant feature was complex treatment regimens (Q11). Furthermore, the lengthy duration of the cutaneous lesion (Q12), the variables opposing effects of treatment (Q13), cultural and lay beliefs about illness and treatment (Q8), life stress, hopelessness, and negative feelings (Q22) were the other most common significant variables in the two analysis approaches. Providing necessary knowledge about the disease and treatment of patients with chronic diseases and patients with misconceptions about chemical treatments are critical issues directly associated with the disease's faithfulness. Moreover, early detection of cases to prevent the extended duration of the disease and the process of treatment, efforts of medical staff and physicians to minimize side effects of treatment, induction of positive thinking, and giving hope to patients with stress and anxiety by medical staff and family can further help patients adhere the treatment.

## Funding

This study was supported by the Vice-Chancellor of Research, 10.13039/501100004621Kerman University of Medical Sciences Kerman, Iran (contract no. 98000240).

## Data availability statement

Data will be made available upon request.

## Declaration of competing interest

The authors declare that they have no known competing financial interests or personal relationships that could have appeared to influence the work reported in this paper.
